# Supplementary material for: Diversity of hydrodynamic radii of intrinsically disordered proteins
Source: Eur Biophys J. 2023 Oct 13;52(6-7):607–18. doi: 10.1007/s00249-023-01683-8 (PMC10618399; doi:10.1007/s00249-023-01683-8)
Supplement: Supplementary file 1 — Supplementary file1 (DOCX 159 KB) [file 249_2023_1683_MOESM1_ESM.docx]

**Supplementary information**

**Diversity of hydrodynamic radii of intrinsically disordered proteins**

Michał K. Białobrzewski^1^, Barbara P. Klepka^1^, Agnieszka Michaś^1^, Maja K. Cieplak-Rotowska^1,2,3^, Zuzanna Staszałek^1^, Anna Niedźwiecka^1,^*

*^1^ Laboratory of Biological Physics, Institute of Physics, Polish Academy of Sciences, Aleja Lotnikow 32/46, PL-02668, Warsaw, Poland*

*^2^ Division of Biophysics, Institute of Experimental Physics, Faculty of Physics, University of Warsaw, Pasteura 5, PL-02093, Warsaw, Poland*

*^3^ present address: The International Institute of Molecular Mechanisms and Machines, Polish Academy of Sciences, Flisa 6, PL-02247 Warsaw, Poland*

*) corresponding author: [annan@ifpan.edu.pl](mailto:annan@ifpan.edu.pl)

**Data of own protein constructs studied in this work**

Protein name, schematic sequence composition and full sequence

*Intrinsically disordered proteins*

**CNOT1 M long**

linker-CNOT1(728-1267)-His_6_

GPLGSPEFPG RPMASMTGGQ QMGGSGSGSP HTQSMQGFPP NLGSAFSTPQ SPAKAFPPLS

TPNQTTAFSG IGGLSSQLPV XGLGTGSLTG IGTGALGLPA VNNDPFVQRK LGTSGLNQPT

FQQTDLSQVW PEANQHFSKE IDDEANSYFQ RIYNHPPHPT MSVDEVLEML XRFKDSTIKR

EREVFNCMLR NLFEEYRFFP QYPDKELHIT ACLFGGIIEK GLVTYMALGL ALRYVLEALR

KPFGSKMYYF GIAALDRFKN RLKDYPQYCQ HLASISHFMQ FPHHLQEYIE YGQQSRDPPV

KMQGSITTPG SIALAQAQAQ AQVPAKAPLA GQVSTMVTTS TTTTVAKTVT VTRPTGVSFK

KDVPPSINTT NIDTLLVATD QTERIVEPPE NIQEKIAFIF NNLSQSNMTQ KVEELKETVK

EEFMPWVSQY LVMKRVSIEP NFHSLYSNFL DTLKNPEFNK MVXNETYRNI KVLLTXDKAA

ANFSDRSLLK NLGHWLGMIT LAKNKPILHT DLDVKSLLLE AYVKGQQELX YVVPFVAKVL

ESSIRSVVFR PPNPWTMAIM NVLAELHQEH HHHHH

**SUMO-GW182 SD**

His_6_-SUMO-linker-GW182(1260-1690)

MGSSHHHHHH SSGLVPRGSH MASMSDSEVN QEAKPEVKPE VKPETHINLK VSDGSSEIFF
KIKKTTPLRR LMEAFAKRQG KEMDSLRFLY DGIRIQADQT PEDLDMEDND IIEAHREQIG
GSEFNTFAPY PLAGLNPNMN VNSMDMTGGL SVKDPSQSQS RLPQWTHPNS MDNLPSAASP
LEQNPSKHGA IPGGLSIGPP GKSSIDDSYG RYDLIQNSES PASPPVAVPH SWSRAKSDSD
KISNGSSINW PPEFHPGVPW KGLQNIDPEN DPDVTPGSVP TGPTINTTIQ DVNRYLLKSG
GKLSDIKSTW SSGPTSHTQA SLSHELWKVP RNSTAPTRPP PGLTNPKPSS TWGASPLGWT
SSYSSGSAWS TDTSGRTSSW LVLRNLTPQI DGSTLRTLCL QHGPLITFHL NLTQGNAVVR
YSSKEEAAKA QKSLHMCVLG NTTILAEFAG EEEVNRFLAQ GQALPPTSSW QSSSASSQPR
LSAAGSSHGL VRSDAGHWNA PCLGGKGSSE LLWGGVPQYS SSLWGPPSAD DSRVIGSPTP
LTTLLPGDLL SGESL

**SUMO-GW182 SD10**

His_6_-SUMO-linker-GW182SD10(1260-1620)-FLAG

MGSSHHHHHH SSGLVPRGSH MASMSDSEVN QEAKPEVKPE VKPETHINLK VSDGSSEIFF
KIKKTTPLRR LMEAFAKRQG KEMDSLRFLY DGIRIQADQT PEDLDMEDND IIEAHREQIG
GSEFNTFAPY PLAGLNPNMN VNSMDMTGGL SVKDPSQSQS RLPQWTHPNS MDNLPSAASP
LEQNPSKHGA IPGGLSIGPP GKSSIDDSYG RYDLIQNSES PASPPVAVPH SWSRAKSDSD
KISNGSSINW PPEFHPGVPW KGLQNIDPEN DPDVTPGSVP TGPTINTTIQ DVNRYLLKSG
GKLSDIKSTW SSGPTSHTQA SLSHELWKVP RNSTAPTRPP PGLTNPKPSS TWGASPLGWT
SSYSSGSAWS TDTSGRTSSW LVLRNLTPQI DGSTLRTLCL QHGPLITFHL NLTQGNAVVR
YSSKEEAAKA QKSLHMCVLG NTTILAEFAG EEEVNRFLAQ GQALPPTSSW QSSSASSQPR
LSAAGADYKD DDDK

**PARN C-mCherry**

PARN(500-639)-linker-mCherry

YAESYRIQTY AEYMGRKQEE KQIKRKWTED SWKEADSKRL NPQCIPYTLQ NHYYRNNSFT

APSTVGKRNL SPSQEEAGLE DGVSGEISDT ELEQTDSCAE PLSEGRKKAK KLKRMKKELS

PAGSISKNSP ATLFEVPDTW LEVLFQGPGS AGSAAGSGEF VSKGEEDNMA IIKEFMRFKV

HMEGSVNGHE FEIEGEGEGR PYEGTQTAKL KVTKGGPLPF AWDILSPQFM YGSKAYVKHP

ADIPDYLKLS FPEGFKWERV MNFEDGGVVT VTQDSSLQDG EFIYKVKLRG TNFPSDGPVM

QKKTMGWEAS SERMYPEDGA LKGEIKQRLK LKDGGHYDAE VKTTYKAKKP VQLPGAYNVN

IKLDITSHNE DYTIVEQYER AEGRHSTGGM DELYK

**GW182 SD10**

linker-GW182SD10(1260-1620)-FLAG

SEFNTFAPYP LAGLNPNMNV NSMDMTGGLS VKDPSQSQSR LPQWTHPNSM DNLPSAASPL
EQNPSKHGAI PGGLSIGPPG KSSIDDSYGR YDLIQNSESP ASPPVAVPHS WSRAKSDSDK
ISNGSSINWP PEFHPGVPWK GLQNIDPEND PDVTPGSVPT GPTINTTIQD VNRYLLKSGG
KLSDIKSTWS SGPTSHTQAS LSHELWKVPR NSTAPTRPPP GLTNPKPSST WGASPLGWTS
SYSSGSAWST DTSGRTSSWL VLRNLTPQID GSTLRTLCLQ HGPLITFHLN LTQGNAVVRY
SSKEEAAKAQ KSLHMCVLGN TTILAEFAGE EEVNRFLAQG QALPPTSSWQ SSSASSQPRL
SAAGADYKDD DDK

**SUMO-GW182 SD peptide**

His_6_-SUMO-linker-GW182(1320-1366)

MGSSHHHHHH SSGLVPRGSH MASMSDSEVN QEAKPEVKPE VKPETHINLK VSDGSSEIFF

KIKKTTPLRR LMEAFAKRQG KEMDSLRFLY DGIRIQADQT PEDLDMEDND IIEAHREQIG

GSEFPSKHGA IPGGLSIGPP GKSSIDDSYG RYDLIQNSES PASPPVAVPH S

*Globular protein*

**CNOT1 M short**

linker-CNOT1(800-999)

SEFNNDPFVQ RKLGTSGLNQ PTFQQTDLSQ VWPEANQHFS KEIDDEANSY FQRIYNHPPH

PTMSVDEVLE MLQRFKDSTI KREREVFNCM LRNLFEEYRF FPQYPDKELH ITACLFGGII

EKGLVTYMAL GLALRYVLEA LRKPFGSKMY YFGIAALDRF KNRLKDYPQY CQHLASISHF

MQFPHHLQEY IEYGQQSRDP PVK

**Supplementary Table S1.**

Data of intrinsically disordered proteins collected from literature and shown in Figure 3.

| **No.** | **Protein** | **N (res)** | **M (Da)** | **M^1/3^ (Da^1/3^)** | **R_h_ (Å)** | **Method** | **Varying conditions** | **Ref.** |
| --- | --- | --- | --- | --- | --- | --- | --- | --- |
| 1 | Aβ(12–24) | 13 | 1569 | 11.62 | 10.6 | PFG-NMR |  | (Danielsson et al. 2002) |
| 2 | Aβ(12–28)G_19_G_20_ | 17 | 1775 | 12.11 | 11.0 | PFG-NMR |  | (Danielsson et al. 2002) |
| 3 | Aβ(12–28)E22G | 17 | 1883 | 12.35 | 11.5 | PFG-NMR |  | (Danielsson et al. 2002) |
| 4 | Aβ(12–28)D23G | 17 | 1897 | 12.38 | 11.1 | PFG-NMR |  | (Danielsson et al. 2002) |
| 5 | Aβ(12–28)D23A | 17 | 1911 | 12.41 | 11.2 | PFG-NMR |  | (Danielsson et al. 2002) |
| 6 | Aβ(12–28)E22D | 17 | 1941 | 12.47 | 11.3 | PFG-NMR |  | (Danielsson et al. 2002) |
| 7 | Aβ(12–28)E22Q | 17 | 1954 | 12.50 | 11.2 | PFG-NMR |  | (Danielsson et al. 2002) |
| 8 | Aβ(12–28)E22K | 17 | 1954 | 12.50 | 11.5 | PFG-NMR |  | (Danielsson et al. 2002) |
| 9 | Aβ(12–28)D23K | 17 | 1968 | 12.53 | 11.5 | PFG-NMR |  | (Danielsson et al. 2002) |
| 10 | Aβ(12–28)D23E | 17 | 1969 | 12.53 | 11.3 | PFG-NMR |  | (Danielsson et al. 2002) |
| 11 | Aβ(1–28) | 28 | 3263 | 14.83 | 14.6 | PFG-NMR |  | (Danielsson et al. 2002) |
| 12 | Aβ(1–40) | 40 | 4330 | 16.30 | 16.1 | PFG-NMR |  | (Danielsson et al. 2002) |
| 13 | Smad binding domain (SBD) | 61 | 6264 | 18.43 | 25.6 | PFG-NMR |  | (Chong et al. 2004) |
| 14 | EHD-L16A | 61 | 7408 | 19.49 | 20.1 | AUC |  | (Mayor et al. 2003) |
| 15 | p57-ID | 71 | 8271 | 20.22 | 24.0 | SEC |  | (Adkins and Lumb 2002) |
| 16 | p53-TAD | 73 | 8205 | 20.17 | 23.8 | SEC |  | (Lowry et al. 2008) |
| 17 | Ntr2^1-75^ | 75 | 8418 | 20.34 | 21.0 | DLS |  | (Wollenhaupt et al. 2018) |
| 18 | PDE-γ | 87 | 9669 | 21.30 | 24.8 | SEC |  | (Uversky et al. 2002b) |
| 19 | C-term-Vmw65 | 89 | 9330 | 21.05 | 28.0 | SEC |  | (Donaldson and Capone 1992) |
| 20 | p53(1-93) | 93 | 9979 | 21.53 | 32.8 | DLS |  | (Perez et al. 2014) |
| 21 | p53(1-93) ALA^-^ | 93 | 9811 | 21.41 | 31.1 | DLS |  | (Perez et al. 2014) |
| 22 | p53(1-93) PRO^-^ | 93 | 9098 | 20.88 | 27.5 | DLS |  | (Perez et al. 2014) |
| 23 | p53(1-93) PRO^-^ ALA^-^ | 93 | 8929 | 20.75 | 27.7 | DLS |  | (Perez et al. 2014) |
| 24 | p53(1-93) | 93 | 9979 | 21.53 | 32.0 | SEC |  | (Perez et al. 2014) |
| 25 | p53(1-93) ALA^-^ | 93 | 9811 | 21.41 | 30.4 | SEC |  | (Perez et al. 2014) |
| 26 | p53(1-93) PRO^-^ | 93 | 9098 | 20.88 | 27.4 | SEC |  | (Perez et al. 2014) |
| 27 | p53(1-93) PRO^-^ ALA^-^ | 93 | 8929 | 20.75 | 27.4 | SEC |  | (Perez et al. 2014) |
| 28 | p53(1-93) | 93 | 9979 | 21.53 | 35.0 | DLS | 5 °C | (Langridge et al. 2014) |
| 29 | p53(1-93) | 93 | 9979 | 21.53 | 26.0 | DLS | 75 °C | (Langridge et al. 2014) |
| 30 | E_m_ protein | 93 | 9963 | 21.52 | 28.2 | SEC |  | (McCubbin et al. 1985) |
| 31 | Mlph(147-240) | 94 | 10111 | 21.62 | 28.0 | SEC |  | (Geething and Spudich 2007) |
| 32 | Hdm2-ABD | 97 | 10723 | 22.05 | 25.7 | Hdm2-ABD |  | (Sivakolundu et al. 2008) |
| 33 | Sml1 | 104 | 11834 | 22.79 | 23.4 | PFG-NMR |  | (Danielsson et al. 2008) |
| 34 | C-terminal fragment TyrRS(∆4) | 107 | 11908 | 22.84 | 21.0 | SEC |  | (Guez et al. 2000) |
| 35 | His-Ek-LjIDP1 | 108 | 11680 | 22.69 | 24.5 | SEC |  | (Haaning et al. 2008) |
| 36 | prothymosin α (1) | 109 | 11985 | 22.88 | 31.4 | SEC | pH 7.5 | (Uversky et al. 1999) |
| 37 | prothymosin α (1) | 109 | 11985 | 22.88 | 24.9 | SEC | pH 2.2 | (Uversky et al. 1999) |
| 38 | prothymosin α (2) | 110 | 12074 | 22.94 | 33.7 | PFG-NMR | no Zn^2+^ | (Yi et al. 2007) |
| 39 | prothymosin α (2) | 110 | 12074 | 22.94 | 24.1 | PFG-NMR | nZn^2+^/nProTα = 30 | (Yi et al. 2007) |
| 40 | ASR1 | 115 | 13130 | 23.59 | 27.4 | SEC |  | (Goldgur et al. 2007) |
| 41 | Nup116 FG Domain | 126 | 12613 | 23.28 | 25.2 | SEC |  | (Krishnan et al. 2008) |
| 42 | Nup116 FG Domain F>A | 126 | 11852 | 22.80 | 27.1 | SEC |  | (Krishnan et al. 2008) |
| 43 | TC1 | 126 | 14714 | 24.50 | 26.5 | PFG-NMR |  | (Gall et al. 2007) |
| 44 | γ-synuclein | 127 | 13331 | 23.71 | 30.4 | SEC | pH 7.5, 100 mM NaCl | (Uversky et al. 2002a) |
| 45 | γ-synuclein | 127 | 13331 | 23.71 | 26.5 | SEC | pH 3.0, 100 mM NaCl | (Uversky et al. 2002a) |
| 46 | AaFEcR | 131 | 13295 | 23.69 | 27.7 | SEC | nM Ca^2+^/nAaFEcR = 0.05 | (Więch et al. 2019) |
| 47 | AaFEcR | 131 | 13295 | 23.69 | 24.4 | SEC | nM Zn^2+^/nAaFEcR = 2.5 | (Więch et al. 2019) |
| 48 | ß-synuclein | 134 | 14288 | 24.27 | 33.9 | SEC | pH 7.5, 100 mM NaCl | (Uversky et al. 2002a) |
| 49 | ß-synuclein | 134 | 14288 | 24.27 | 27.5 | SEC | pH 3.0, 100 mM NaCl | (Uversky et al. 2002a) |
| 50 | Pro/Gly-rich region of Accumulation-associated protein (Aap PGR) | 135 | 13139 | 23.60 | 37.1 | SEC | 22 °C | (Yarawsky et al. 2017) |
| 51 | Pro/Gly-rich region of Accumulation-associated protein (Aap PGR) | 135 | 13139 | 23.60 | 38.4 | DLS | 25 °C (no significant changes in R_h_ in the temperature range of  5 °C to 45 °C | (Yarawsky et al. 2017) |
| 52 | CaD136 | 136 | 14427 | 24.34 | 28.1 | SEC |  | (Permyakov et al. 2003) |
| 53 | N_TAIL_ | 139 | 15399 | 24.88 | 27.0 | SEC |  | (Longhi et al. 2003) |
| 54 | N_TAIL_ | 139 | 15399 | 24.88 | 30.0 | DLS |  | (Longhi et al. 2003) |
| 55 | α-synuclein | 140 | 14460 | 24.36 | 28.2 | PFG-NMR |  | (Paleologou et al. 2008) |
| 56 | S87A α-synuclein | 140 | 14444 | 24.35 | 28.1 | PFG-NMR |  | (Paleologou et al. 2008) |
| 57 | P Ser-129 Ser-87  α-synuclein | 140 | 14616 | 24.45 | 35.3 | PFG-NMR |  | (Paleologou et al. 2008) |
| 58 | P Ser-129 S87A  α-synuclein | 140 | 14522 | 24.40 | 34.7 | PFG-NMR |  | (Paleologou et al. 2008) |
| 59 | α-synuclein | 140 | 14460 | 24.36 | 31.8 | SEC | pH 7.5, 100 mM NaCl | (Uversky et al. 2002a) |
| 60 | α-synuclein | 140 | 14460 | 24.36 | 27.9 | SEC | pH 3.0, 100 mM NaCl | (Uversky et al. 2002a) |
| 61 | hNL3-cyt | 140 | 15290 | 24.82 | 28.3 | SEC |  | (Paz et al. 2008) |
| 62 | hNL3-cyt | 140 | 15290 | 24.82 | 23.3 | DLS |  | (Paz et al. 2008) |
| 63 | hNL3-cyt | 140 | 15290 | 24.82 | 25.0 | FCS |  | (Paz et al. 2008) |
| 64 | hNL3-cyt | 140 | 15290 | 24.82 | 27.3 | AUC |  | (Paz et al. 2008) |
| 65 | ShB-C | 146 | 15912 | 25.15 | 32.9 | SEC |  | (Magidovich et al. 2007) |
| 66 | Ntr2^1-162^ | 162 | 18141 | 26.28 | 31.0 | DLS |  | (Wollenhaupt et al. 2018) |
| 67 | Fos-AD | 168 | 17612 | 26.02 | 35.0 | SEC |  | (Campbell et al. 2000) |
| 68 | HIF-1α (530-698) | 170 | 18755 | 26.57 | 38.3 | SEC |  | (Sánchez-Puig et al. 2005a) |
| 69 | CFTR R region | 189 | 21410 | 27.77 | 32.0 | PFG-NMR |  | (Baker 2009) |
| 70 | Tau K32 | 198 | 21030 | 27.60 | 45.0 | PFG-NMR |  | (Soragni et al. 2008) |
| 71 | HIF-1α (403-603) | 201 | 22157 | 28.09 | 44.3 | SEC |  | (Sánchez-Puig et al. 2005a) |
| 72 | DARPP-32 | 202 | 22614 | 28.28 | 34.0 | SEC |  | (Hemmings et al. 1984) |
| 73 | Securin | 204 | 22247 | 28.12 | 39.7 | SEC |  | (Sánchez-Puig et al. 2005b) |
| 74 | SNAP25 | 206 | 23315 | 28.57 | 39.3 | DLS |  | (Choi et al. 2011) |
| 75 | Gliotactin-cyt | 217 | 23594 | 28.68 | 33.4 | SEC |  | (Zeev-Ben-Mordehai et al. 2003) |
| 76 | 3D7-6H MSP2 | 237 | 24191 | 28.92 | 41.0 | PFG-NMR | pH 3.6; 10 mM HOAc | (Zhang et al. 2008) |
| 77 | 3D7-6H MSP2 | 237 | 24191 | 28.92 | 34.3 | PFG-NMR | pH 7.0; PBS | (Zhang et al. 2008) |
| 78 | 3D7-6H MSP2 | 237 | 24191 | 28.92 | 34.9 | PFG-NMR | pH 3.5; 10 mM HOAc + 135 mM NaCl | (Zhang et al. 2008) |
| 79 | RYBP | 234 | 25644 | 29.49 | 39.5 | PFG-NMR |  | (Neira et al. 2009) |
| 80 | H_6_-PNT | 236 | 24849 | 29.18 | 41.0 | SEC |  | (Karlin et al. 2002) |
| 81 | H_6_-PNT | 236 | 24849 | 29.18 | 47.0 | DLS |  | (Karlin et al. 2002) |
| 82 | Mlph(147-403) | 260 | 27903 | 30.33 | 46.0 | DLS |  | (Geething and Spudich 2007) |
| 83 | Mlph(147-403) | 260 | 27903 | 30.33 | 49.0 | SEC |  | (Geething and Spudich 2007) |
| 84 | Ntr2^FL^ | 322 | 36647 | 33.22 | 42.0 | DLS |  | (Wollenhaupt et al. 2018) |
| 85 | DBE | 345 | 39843 | 34.15 | 33.1 | SEC |  | (Yiu et al. 2006) |
| 86 | Calreticulin | 404 | 46791 | 36.03 | 46.2 | SEC |  | (Bouvier and Stafford 2000) |
| 87 | HeV PNT | 410 | 45216 | 35.63 | 44.0 | SEC |  | (Habchi et al. 2010) |
| 88 | HeV PNT | 410 | 45216 | 35.63 | 50.0 | DLS |  | (Habchi et al. 2010) |
| 89 | NiV PNT | 412 | 45331 | 35.66 | 44.0 | SEC |  | (Habchi et al. 2010) |
| 90 | NiV PNT | 412 | 45331 | 35.66 | 44.0 | DLS |  | (Habchi et al. 2010) |
| 91 | Nup159pΔNΔC | 441 | 44698 | 35.49 | 61.0 | SEC |  | (Denning et al. 2003) |
| 92 | Mid1p-N452 | 458 | 50503 | 36.96 | 49.0 | SEC |  | (Chatterjee and Pollard 2019) |
| 93 | Starmaker | 593 | 64043 | 40.01 | 78.6 | SEC |  | (Kapłon et al. 2008) |
| 94 | Nsp1pΔC | 603 | 61887 | 39.55 | 74.0 | SEC |  | (Denning et al. 2003) |
| 95 | OMM-64 | 608 | 64473 | 40.10 | 76.2 | SEC |  | (Poznar et al. 2017) |
| 96 | OMM-64 | 608 | 64473 | 40.10 | 74.5 | FCS |  | (Poznar et al. 2017) |
| 97 | OMM-64 | 608 | 64473 | 40.10 | 75.9 | AUC |  | (Poznar et al. 2017) |
| 98 | OMM-64 | 608 | 64473 | 40.10 | 85.0 | SEC | no salt | (Poznar et al. 2017) |
| 99 | OMM-64 | 608 | 64473 | 40.10 | 70.0 | SEC | 100 mM CaCl_2_ | (Poznar et al. 2017) |
| 100 | Nup100pΔC | 640 | 64454 | 40.09 | 59.0 | SEC |  | (Denning et al. 2003) |
| 101 | Nup2p | 720 | 77881 | 42.70 | 79.0 | SEC |  | (Denning et al. 2002) |
| 102 | Nup85p | 744 | 84898 | 43.95 | 51.0 | SEC |  | (Denning et al. 2003) |
| 103 | Caldesmon | 771 | 88747 | 44.61 | 91.0 | SEC |  | (Lynch et al. 1987) |
| 104 | Nup1pΔN | 777 | 80671 | 43.21 | 75.0 | SEC |  | (Denning et al. 2003) |
| 105 | Fesselin | 996 | 107712 | 47.58 | 53.0 | SEC |  | (Khaymina et al. 2007) |

**Sequences of proteins gathered in Table S1.**

**Aβ(12–24)**

VHHQKLVFFA EDV

**Aβ(12–28)G_19_G_20_**

VHHQKLVGGA EDVGSNK

**Aβ(12–28)E22G**

VHHQKLVFFA GDVGSNK

**Aβ(12–28)D23G**

VHHQKLVFFA EGVGSNK

**Aβ(12–28)D23A**

VHHQKLVFFA EAVGSNK

**Aβ(12–28)E22D**

VHHQKLVFFA DDVGSNK

**Aβ(12–28)E22Q**

VHHQKLVFFA QDVGSNK

**Aβ(12–28)E22K**

VHHQKLVFFA KDVGSNK

**Aβ(12–28)D23K**

VHHQKLVFFA EKVGSNK

**Aβ(12–28)D23E**

VHHQKLVFFA EEVGSNK

**Aβ(1–28)**

DAEFRHDSGY EVHHQKLVFF AEDVGSNK

**Aβ(1–40)**

DAEFRHDSGY EVHHQKLVFF AEDVGSNKGA IIGLMVGGVV

**Smad binding domain (SBD)**

GSMMSASSQS PNPNNPAEYC STIPPLEYCS TIPPLQQAQA SGALSSPPPT VMVPVGVLKH P

**EHD-L16A**

TNDEKRPRTA FSSEQAARLK REFNENRYLT ERRRQQLSSE LGLNEAQIKI WFQNKRAKIK K

**p57-ID**

TSACRSLFGP VDHEELSREL QARLAELNAE DQNRWDYDFQ QDMPLRGPGR LQWTEVDSDS VPAFYRETVQ V

**p53-TAD**

MEEPQSDPSV EPPLSQETFS DLWKLLPENN VLSPLPSQAM DDLMLSPDDI EQWFTEDPGP DEAPRMPEAA PRV

**Ntr2^1-75^**

MAIKKRNKIR LPSGSPEEVG IDGSAHKPMQ QIKPLVSNDS EDDDNDICVL QPIKFKKVPK RDITFDGEQA IKEDN

**PDE-γ**

MNLEPPKAEI RSATRVMGGP VTPRKGPPKF KQRQTRQFKS KPPKKGVQGF GDDIPGMEGL GTDITVICPW EAFNHLELHE LAQYGII

**C-term-Vmw65**

GSAGHTRRLS TAPPTDVSLG DELHLDGEDV AMAHADALDD FDLDMLGDGD SPGPGFTPHD SAPYGALDMA DFEFEQMFTD ALGIDEYGG

**p53(1-93)**

MEEPQSDPSV EPPLSQETFS DLWKLLPENN VLSPLPSQAM DDLMLSPDDI EQWFTEDPGP DEAPRMPEAA PPVAPAPAAP TPAAPAPAPS WPL

**p53(1-93) ALA-**

MEEPQSDPSV EPPLSQETFS DLWKLLPENN VLSPLPSQGM DDLMLSPDDI EQWFTEDPGP DEGPRMPEGG PPVGPGPGGP TPGGPGPGPS WPL

**p53(1-93) PRO-**

MEEGQSDGSV EGGLSQETFS DLWKLLGENN VLSGLGSQAM DDLMLSGDDI EQWFTEDGGG DEAGRMGEAA GGVAGAGAAG TGAAGAGAGS WGL

**p53(1-93) ALA- PRO-**

MEEGQSDGSV EGGLSQETFS DLWKLLGENN VLSGLGSQGM DDLMLSGDDI EQWFTEDGGG DEGGRMGEGG GGVGGGGGGG TGGGGGGGGS WGL

**E_m_ protein**

MASGQQERSQ LDRKAREGET VVPGGTGGKS LEAQENLAEG RSRGGQTRRE QMGEEGYSQM GRKGGLSTND ESGGDRAARE GIDIDESKFK TKS

**Mlph(147-240)**

GGGGSEPSLE EGNGDSEQTD EDGDLDTEAR DQPLNSKKKK RLLSFRDVDF EEDSDHLVQP CSQTLGLSSV PESAHSLQSL SGEPYSEDTT SLEP

**Hdm2-ABD**

XXSSSSESTG TPSNPDLDAG VSEHSGDWLD QDSVSDQFSV EFEVESLDSE DYSLSEEGQE

LSDEDDEVYQ VTVYQAGESD TDSFEEDPEI SLADYWK

**Sml1**

MQNSQDYFYA QNRCQQQQAP STLRTVTMAE FRRVPLPPMA EVPMLSTQNS MGSSASASAS SLEMWEKDLE ERLNSIDHDM NNNKFGSGEL KSMFNQGKVE EMDF

**C-terminal fragment TyrRS(∆4)**

ALFSGDIANL TAAEIEQGFK DVPSFVHEGG DVPLVELLVS AGISPSKRQA REDIQNGAIY VNGERLQDVG AILTAEHRLE GRFTVIRRGK KKYYLIRYAL GHHHHHH

**His-Ek-LjIDP1**

MAHHHHHHVD DDDKMARSFT NIKAISALVA EEFSNSLARR GYAATAQSAG RVGASMSGKM

GSTKSGEEKA AAREKVSWVP DPVTGYYKPE NIKEIDVAEL RSAVLGKN

**prothymosin α (1)**

SDAAVDTSSE ITTKDLKEKK EVVEEAENGR DAPANGNANE ENGEQEADNE VDEEEEEGGE

EEEEEEEGDG EEEDVDEDEE AESATGKRAA EDDEDDDVDT KKQKTDEDD

**prothymosin α (2)**

MSDAAVDTSS EITTKDLKEK KEVVEEAENG RDAPANGNAN EENGEQEADN EVDEEEEEGG

EEEEEEEEGD GEEEDGDEDE EAESATGKRA AEDDEDDDVD TKKQKTDEDD

**ASR1**

MEEEKHHHHH LFHHKDKAEE GPVDYEKEIK HHKHLEQIGK LGTVAAGAYA LHEKHEAKKD

PEHAHKHKIE EEIAAAAAVG AGGFAFHEHH EKKDAKKEEK KKLRGDTTIS SKLLF

**Nup116 FG Domain**

GSRRASVGSG ALFGAKPASG GLFGQSAGSK AFGMNTNPTG TTGGLFGQTN QQQSGGGLFG

QQQNSNAGGL FGQNNQSQNQ SGLFGQQNSS NAFGQPQQQG GLFGSKPAGG LFGQQQGAST

HHHHHH

**Nup116 FG Domain F>A**

GSRRASVGSG ALAGAKPASG GLAGQSAGSK AAGMNTNPTG TTGGLAGQTN QQQSGGGLAG

QQQNSNAGGL AGQNNQSQNQ SGLAGQQNSS NAAGQPQQQG GLAGSKPAGG LAGQQQGAST

HHHHHH

**TC1**

HHHHHHXXXX XXXXXXXXXX MKAKRSHQAI IMSTSLRVSP SIHGYHFDTA SRKKAVGNIF
ENTDQESLER LFRNSGDKKA EERAKIIFAI DQDVEEKTRA LMALKKRTKD KLFQFLKLRK
YSIKVH

**γ-synuclein**

MDVFKKGFSI AKEGVVGAVE KTKQGVTEAA EKTKEGVMYV GAKTKENVVQ SVTSVAEKTK

EQANAVSEAV VSSVNTVATK TVEEAENIAV TSGVVRKEDL RPSAPQQEGE ASKEKEEVAE

EAQSGGD

**AaFEcR**

GPSAGLVPRG SGGIEGRHML EEIWDVQDIP PSMQAQMHSH GTQSSSSSSS SSSSSSNGSS

NGNSSSNSNS SQHGPHPHPH GQQLTPNQQQ HQQQHSQLQQ VHANGSGSGG GSNNNSSSGG

VVPGLGMLDQ V

**β-synuclein**

MDVFMKGLSM AKEGVVAAAE KTKQGVTEAA EKTKEGVLYV GSKTREGVVQ GVASVAEKTK

EQASHLGGAV FSGAGNIAAA TGLVKREEFP TDLKPEEVAQ EAAEEPLIEP LMEPEGESYE

DPPQEEYQEY EPEA

**Proline/glycine-rich region (PGR) of Accumulation-associated protein (Aap)**

AEPGKPAEPG KPAEPGKPAE PGTPAEPGKP AEPGTPAEPG KPAEPGKPAE PGKPAEPGKP

AEPGTPAEPG TPAEPGKPAE PGTPAEPGKP AEPGTPAEPG KPAESGKPVE PGTPAQSGAP

EQPNRSMHST DNKNQ

**CaD136**

RLEQYTSAVV GNKAAKPAKP AASDLPVPAE GVRNIKSMWE KGNVFSSPGG TGTPNKETAG

LKVGVSSRIN EWLTKTPEGN KSPAPKPSDL RPGDVSGKRN LWEKQSVEKP AASSSKVTAT

GKKSETNGLR QFEKEP

**N_TAIL_**

MRGSHHHHHH XXXHTTEDKI SRAVGPRQAQ VSFLHGDQSE NELPRLGGKE DRRVKQSRGE

ARESYRETGP SRASDARAAH LPTGTPLDID TASESSQDPQ DSRRSADALL RLQAMAGISE

EQGSDTDTPI VYNDRNLLD

**α-synuclein**

MDVFMKGLSK AKEGVVAAAE KTKQGVAEAA GKTKEGVLYV GSKTKEGVVH GVATVAEKTK

EQVTNVGGAV VTGVTAVAQK TVEGAGSIAA ATGFVKKDQL GKNEEGAPQE GILEDMPVDP

DNEAYEMPSE EGYQDYEPEA

**S87A α-synuclein**

MDVFMKGLSK AKEGVVAAAE KTKQGVAEAA GKTKEGVLYV GSKTKEGVVH GVATVAEKTK

EQVTNVGGAV VTGVTAVAQK TVEGAGAIAA ATGFVKKDQL GKNEEGAPQE GILEDMPVDP

DNEAYEMPSE EGYQDYEPEA

**hNL3-cyt**

MGSSHHHHHH SSGLVPRGSH MAYRKDKRRQ EPLRQPSPQR GAGAPELGAA PEEELAALQL

GPTHHECEAG PPHDTLRLTA LPDYTLTLRR SPDDIPLMTP NTITMIPNSL VGLQTLHPYN

TFAAGFNSTG LPHSHSTTRV

**ShB-C**

MXXGQHMKKS SLSESSSDMM DLDDGVESTP GLTETHPGRS AVAPFLGAQQ QQQQPVASSL

SMSIDKQLQH PLQQLTQTQL YQQQQQQQQQ QQNGFKQQQQ QTQQQLQQQQ SHTINASAAA

ATSGSGSSGL TMRHNNALAV SIETDV

**Ntr2^1-162^**

MAIKKRNKIR LPSGSPEEVG IDGSAHKPMQ QIKPLVSNDS EDDDNDICVL QPIKFKKVPK

RDITFDGEQA IKEDNSHYED LYHSKKNTNA STRNKDDLLI LNMEDLMEGN HHLLSDSSEA

GSSSEGEHIS SIPTRGEIAK LKAQKSLSRR KISESDVTTE RD

**Fos-AD**

GSHMSVASLD LTGGLPEVAT PESEEAFTLP LLNDPEPKPS VEPVKSISSM ELKTEPFDDF

LFPASSRPSG SETARSVPDM DLSGSFYAAD WEPLHSGSLG MGPMATELEP LCTPVVTCTP

SCTAYTSSFV FTYPEADSFP SCAAAHRKGS SSNEPSSDSL SSPTLLAL

**HIF-1α (530-698)**

XEFKLELVEK LFAEDTEAKN PFSTQDTDLD LEMLAPYIPM DDDFQLRSFD QLSPLESSSA

SPESASPQST VTVFQQTQIQ EPTANATTTT ATTDELKTVT KDRMEDIKIL IASPSPTHIH

KETTSATSSP YRDTQSRTAS PNRAGKGVIE QTEKSHPRSP NVLSVALSQR

**CFTR R region**

GAMESAERRN SILTETLHRF SLEGDAPVSW TETKKQSFKQ TGEFGEKRKN SILNPINSIR

KFSIVQKTPL QMNGIEEDSD EPLERRLSLV PDSEQGEAIL PRISVISTGP TLQARRRQSV

LNLMTHSVNQ GQNIHRKTTA STRKVSLAPQ ANLTELDIYS RRLSQETGLE ISEEINEEDL

KECLFDDME

**Tau K32**

MSSPGSPGTP GSRSRTPSLP TPPTREPKKV AVVRTPPKSP SSAKSRLQTA PVPMPDLKNV

KSKIGSTENL KHQPGGGKVQ IINKKLDLSN VQSKCGSKDN IKHVPGGGSV QIVYKPVDLS

KVTSKCGSLG NIHHKPGGGQ VEVKSEKLDF KDRVQSKIGS LDNITHVPGG GNKKIETHKL

TFRENAKAKT DHGAEIVY

**HIF-1α (403-603)**

AAGDTIISLD FGSNDTETDD QQLEEVPLYN DVMLPSPNEK LQNINLAMSP LPTAETPKPL

RSSADPALNQ EVALKLEPNP ESLELSFTMP QIQDQTPSPS DGSTRQSSPE PNSPSEYCFY

VDSDMVNEFK LELVEKLFAE DTEAKNPFST QDTDLDLEML APYIPMDDDF QLRSFDQLSP

LESSSASPES ASPQSTVTVF Q

**DARPP-32**

MDPKDRKKIQ FSVPAPPSQL DPRQVEMIRR RRPTPAMLFR LSEHSSPEEE ASPHQRASGE

GHHLKSKRSN PCAYTPPSLK AVQRIAESHL QSISNLGENQ ASEEEDELGE LRELGYPREE

EEEEEEEDEE EEEDSQAEVL KGSRGSAGQK TTYGQGLEGP WERPPPLDGP QRDGSSEDQV

EDPALNEPGE EPQRPAHPEP GT

**Securin**

XXMATLIYVD KENGEPGTRV VAKDGLKLGS GPSIKALDGR SQVSTPRFGK TFDAPPALPK

ATRKALGTVN RATEKSVKTK GPLKQKQPSF SAKKMTEKTV KAKSSVPASD DAYPEIEKFF

PFNPLDFESF DLPEEHQIAH LPLSGVPLMI LDEERELEKL FQLGPPSPVK MPSPPWESNL

LQSPSSILST LDVELPPVCC DIDI

**SNAP25**

MAEDADMRNE LEEMQRRADQ LADESLESTR RMLQLVEESK DAGIRTLVML DEQGEQLERI

EEGMDQINKD MKEAEKNLTD LGKFCGLCVC PCNKLKSSDA YKKAWGNNQD GVVASQPARV

VDEREQMAIS GGFIRRVTND ARENEMDENL EQVSGIIGNL RHMALDMGNE IDTQNRQIDR

IMEKADSNKT RIDEANQRAT KMLGSG

**Gliotactin-cyt**

XXXRNAKRQS DRFYDEDVFI NGEGLEPEQD TRGVDNAHMV TNHHALRSRD NIYEYRDSPS

TKTLASKAHT DTTSLRSPSS LAMTQKSSSQ ASLKSGISLK ETNGHLVKQS ERAATPRSQQ

NGSIAKVASP PVEEKRLLQP LSSTPVTQLQ AEPAKRVPTA ASVSGSSRST TPVPSARSTT

THTTTATLSS QPAAQPRRTH LVEGVPQTSV XHHHHHH

**3D7-6H MSP2**

MIKNESKYSN TFINNAYNMS IRRSMAESKP STGAGGSAGG SAGGSAGGSA GGSAGGSAGS

GDGNGADAEG SSSTPATTTT TKTTTTTTTT NDAEASTSTS SENPNHKNAE TNPKGKGEVQ

EPNQANKETQ NNSNVQQDSQ TKSNVPPTQD ADTKSPTAQP EQAENSAPTA EQTESPELQS

APENKGTGQH GHMHGSRNNH PQNTSDSQKE CTDGNKENCG AATSLLNNSS NHHHHHH

**RYBP**

HHHHHHMTMG DKKSPTRPKR QAKPAADEGF WDCSVCTFRN SAEAFKCSIC DVRKGTSTRK

PRINSQLVAQ QVAQQYATPP PPKKEKKEKV EKQDKEKPEK DKEISPSVTK KNTNKKTKPK

SDILKDPPSE ANSIQSANAT TKTSETNHTS RPRLKNVDRS TAQQLAVTVG NVTVIITDFK

EKTRSSSTSS STVTSSAGSE QQNQSSSGSE STDKGSSRSS TPKGDMSAVN DESF

**H_6_-PNT**

HHHHHHMAEE QARHVKNGLE CIRALKAEPI GSLAIEEAMA AWSEISDNPG QERATCREEK

AGSSGLSKPC LSAIGSTEGG APRIRGQGPG ESDDDAETLG IPPRNLQASS TGLQCYYVYD

HSGEAVKGIQ DADSIMVQSG LDGDSTLSGG DNESENSDVD IGEPDTEGYA ITDRGSAPIS

MGFRASDVET AEGGEIHELL RLQSRGNNFP KLGKTLNVPP PPDPGRASTS GTPIKK

**Mlph(147-403)**

XXXGGGGSEP SLEEGNGDSE QTDEDGDLDT EARDQPLNSK KKKRLLSFRD VDFEEDSDHL

VQPCSQTLGL SSVPESAHSL QSLSGEPYSE DTTSLEPEGL EETGARALGC RPSPEVQPCS

PLPSGEDAHA ELDSPAASCK SAFGTTAMPG TDDVRGKHLP SQYLADVDTS DEDSIQGPRA

ASQHSKRRAR TVPETQILEL NKRMSAVEHL LVHLENTVLP PSAQEPTVET HPSADTEEET

LRRRLEELTS NISGSSTSSE

**Ntr2^FL^**

MAIKKRNKIR LPSGSPEEVG IDGSAHKPMQ QIKPLVSNDS EDDDNDICVL QPIKFKKVPK

RDITFDGEQA IKEDNSHYED LYHSKKNTNA STRNKDDLLI LNMEDLMEGN HHLLSDSSEA

GSSSEGEHIS SIPTRGEIAK LKAQKSLSRR KISESDVTTE RDYVKLLDSE DKREIMETIR

LNGGLKRNNE KEITNFSDDE MQGFQDEMLA LTDNQIAIQK DSKRKIIEKA INEVPYRTNE

EWETQLLSKG NINKSNEKII TPLPVLFPDD DESGNSIERI NEMVSKICLQ RKKVEMRLQA

LEKTKIDLEK SKASLINKLI GN

**DBE**

MSESEAEETK ISTEPVDNAW SMKIPAFRQE DNPHGMVEES SFATLFPKYR ERYLKEVWPL

VEQCLAEHHL KAELDLMEGS MVVKTSRKTW DPYIIIKARD MIKLMARSVP FEQAKRVLQD

DIGCDIIKIG NLVHKKEKFV KRRQRLIGPN GATLKSIELL TDCYVLVQGN TVSALGPYKG

LQQVRDIVLE TMNNVHPIYN IKALMIKREL MKDPRLANED WSRFLPKFKN KNISKRKQPK

VKKQKKEYTP FPPSQPESKV DKQLASGEYF LNQEQKQAKR NQERTEKQKE AAKRQDERRN

KDFVPPTEES AASSRKKEDG SSSSKVDVKA LKAKLIKANK KARSS

**Calreticulin**

GIPGEPAVYF KEQFLDGDGW TSRWIESKHK SDFGKFVLSS GKFYGDEEKD KGLQTSQDAR

FYALSASFEP FSNKGQTLVV QFTVKHEQNI DCGGGYVKLF PNSLDQTDMH GDSEYNIMFG

PDICGPGTKK VHVIFNYKGK NVLINKDIRC KDDEFTHLYT LIVRPDNTYE VKIDNSQVES

GSLEDDWDFL PPKKIKDPDA SKPEDWDERA KIDDPTDSKP EDWDKPEHIP DPDAKKPEDW

DEEMDGEWEP PVIQNPEYKG EWKPRQIDNP DYKGTWIHPE IDNPEYSPDP SIYAYDNFGV

LGLDLWQVKS GTIFDNFLIT NDEAYAEEFG NETWGVTKAA EKQMKDKQDE EQRLKEEEED

KKRKEEEEAE DKEDDEDKDE DEEDEEDKEE DEEEDVPGQA KDEL

**HeV PNT**

MDKLDLVNDG LDIIDFIQKN QKEIQKTYGR SSIQQPSTKD RTRAWEDFLQ STSGEHEQAE

GGMPKNDGGT EGRNVEDLSS VTSSDGTIGQ RVSNTRAWAE DPDDIQLDPM VTDVVYHDHG

GECTGHGPSS SPERGWSYHM SGTHDGNVRA VPDTKVLPNA PKTTVPEEVR EIDLIGLEDK

FASAGLNPAA VPFVPKNQST PTEEPPVIPE YYYGSGRRGD LSKSPPRGNV NLDSIKIYTS

DDEDENQLEY EDEFAKSSSE VVIDTTPEDN DSINQEEVVG DPSDQGLEHP FPLGKFPEKE

ETPDVRRKDS LMQDSCKRGG VPKRLPMLSE EFECSGSDDP IIQELEREGS HPGGSLRLRE

PPQSSGNSRN QPDRQLKTGD AASPGGVQRP GTPMPKSRIM PIKKHHHHHH

**NiV PNT**

MDKLELVNDG LNIIDFIQKN QKEIQKTYGR SSIQQPSIKD QTKAWEDFLQ CTSGESEQVE

GGMSKDDGDV ERRNLEDLSS TSPTDGTIGK RVSNTRDWAE GSDDIQLDPV VTDVVYHDHG

GECTGYGFTS SPERGWSDYT SGANNGNVCL VSDAKMLSYA PEIAVSKEDR ETDLVHLENK

LSTTGLNPTA VPFTLRNLSD PAKDSPVIAE HYYGLGVKEQ NVGPQTSRNV NLDSIKLYTS

DDEEADQLEF EDEFAGSSSE VIVGISPEDE EPSSVGGKPN ESIGRTIEGQ SIRDNLQAKD

NKSTDVPGAG PKDSAVKEEP PQKRLPMLAE EFECSGSEDP IIRELLKENS LINCQQGKDA

QPPYHWSIER SISPDKTEIV NGAVQTADRQ RPGTPMPKSR GIPIKKHHHHHH

**Nup159pΔNΔC**

SGFTFLKTQP AAANSLQSQS SSTFGAPSFG SSAFKIDLPS VSSTSTGVAS SEQDATDPAS

AKPVFGKPAF GAIAKEPSTS EYAFGKPSFG APSFGSGKSS VESPASGSAF GKPSFGTPSF

GSGNSSVEPP ASGSAFGKPS FGTPSFGSGN SSAEPPASGS AFGKPSFGTS AFGTASSNET

NSGSIFGKAA FGSSSFAPAN NELFGSNFTI SKPTVDSPKE VDSTSPFPSS GDQSEDESKS

DVDSSSTPFG TKPNTSTKPK TNAFDFGSSS FGSGFSKALE SVGSDTTFKF GTQASPFSSQ

LGNKSPFSSF TKDDTENGSL SKGSTSEIND DNEEHESNGP NVSGNDLTDS TVEQTSSTRL

PETPSDEDGE VVEEEAQKSP IGKLTETIKK SANIDMAGLK NPVFGNHVKA KSESPFSAFA

TNITKPSSTT PAFSFGNSTM N

**Mid1p-N452**

HHHHHHMKEQ EFSYREAKDV SLDSKGLENS FLSSPNREKT PLFFEGNSNE TSGYDQTKNF

THGDGDMSLG NLSELNVATD LLESLDLRSM YMHGYGHLDS SFSSQHSPDN RKRMSSTSVF

KRINSEEEGR IPSLTYSAGT MNSTSSSTAS LKGADIVADY ETFNPDQNLA ELSFDRSKSS

RKRAVEVAEF SRAKTMSPLE YTVQHPYQSH NELSTNPARA RAGSVPNLAR IPSDVKPVPP

AHLSASSTVG PRILPSLPKD TTEDNPALER VETTASLDMD YKPLEPLAPI QEAPVEDTSE

PFSSVPEATL DDSDISTESL RKKVLAKMEA KRISSGSSYA STLRKVYDFS ELSLPTNGKD

YDELYLQSSR NSEPEISTII NDSLQQENMD EDISATSIPK SQAAYGHGSV TYHEVPRYNL

TSASVGYSIS SQRGRIKSSS TIDNLSAILS SEDLRHPS

**Starmaker**

APVSNNNGTD NDESAADQRH IFTVQFNVGT PAPADGDSVT TDGKDSAEKN EAPGDSSDTT

EKPGTTDGKD SAEQHGVTTD GKDEAEQHGV TTDGQDSAEK RGEADGAPDK PDTQNGTDDT

DSDQETDASH HKTGDSDENK DKPSAEDHTD GNHAGKDSTD SKESPDTTDK PEGPDSDSAP

DGDSASAEKT DSDHSPDEDA NKSSTEADKD DTSDKDSSQT DEKHDSDASD KDEKHEDKDE

KSDEKDSSKD SEDKSQEKSD KSDDGSNSEA DEQKESVESK DHDSDSQDSD SAEKKEKHDD

KDQDSSDSAD SKDSDEDKDK DHSEQKDSED HEHKEKHTKD KEEHKDSDEG KDDEDKSKSD

EHDKDESESK EASKSDESEQ EEKKDDKSDS DNSSRDSHSD SDSDSHSDSD SDSHSDSHSD

SDSDSHSDSD SDSDSDSDSD SDSDSDSNSR DKDEKKDKSS ESRDEDSSDS DSKSNSESSE

TAEEDTNDDK DSSVEKDKTD SSDSASVEAN DSDDEHDDDS KDATPSSEDH TAEKTDEDSH

DVSDDDDDID AHDDEAGVEH GTDEASKPHQ EPDHHDDTTH GSDDGRKTSM PIS

**Nsp1pΔC**

MNFNTPQQNK TPFSFGTANN NSNTTNQNSS TGAGAFGTGQ STFGFNNSAP NNTNNANSSI

TPAFGSNNTG NTAFGNSNPT SNVFGSNNST TNTFGSNSAG TSLFGSSSAQ QTKSNGTAGG

NTFGSSSLFN NSTNSNTTKP AFGGLNFGGG NNTTPSSTGN ANTSNNLFGA TANANKPAFS

FGATTNDDKK TEPDKPAFSF NSSVGNKTDA QAPTTGFSFG SQLGGNKTVN EAAKPSLSFG

SGSAGANPAG ASQPEPTTNE PAKPALSFGT ATSDNKTTNT TPSFSFGAKS DENKAGATSK

PAFSFGAKPE EKKDDNSSKP AFSFGAKSNE DKQDGTAKPA FSFGAKPAEK NNNETSKPAF

SFGAKSDEKK DGDASKPAFS FGAKPDENKA SATSKPAFSF GAKPEEKKDD NSSKPAFSFG

AKSNEDKQDG TAKPAFSFGA KPAEKNNNET SKPAFSFGAK SDEKKDGDAS KPAFSFGAKS

DEKKDSDSSK PAFSFGTKSN EKKDSGSSKP AFSFGAKPDE KKNDEVSKPA FSFGAKANEK

KESDESKSAF SFGSKPTGKE EGDGAKAAIS FGAKPEEQKS SDTSKPAFTF GAQKDNEKKT

EES

**OMM-64**

APVNDGTEAD NDERAASLLV HLKGDKDGGG LTGSPDGVSA GTTDGTDSSK ELAGGAVDSS

PDTTDTPDAS SSDIFPDTNN RDTSVETTGN PDDSDAPDAA ESAGSQDTTD AADASEAVAE

TVDTYDIPDT DGADDREKVS TEVSTEDLDS AGVDKSPESD STESPGSDSA ESPGSDSAES

PGSDSTESPG SDSTESPRSD STDEVLTDVQ ADSADVTSDD MDEATETDKD DDKSDDKSDA

DAATDKDDSD EDKDTELDGK AHAEDTQTEE AADSDSKQGA ADSDSDTDDD RPEKDVKNDS

DDSKDTTEDD KPDKDDKKNR DSADNSNDDS DEMIQVPREE LEQQEINLKE GGVIGSQEET

VASDMEEGSD VGDQKPGPED SIEEGSPVGR QDFKHPQDSE EEELEKEAKK EKELEEAEEE

RTLKTIESDS QEDSVDESEA EPDSNSKKDI GTSDAPEPQE DDSEEDTDDS MMKEPKDSDD

AESDKDDKDK NDMDKEDMDK DDMDKDDMDK DDMDKDDVDK DASDSVDDQS ESDAEPGADS

HTVVDEIDGE ETMTPDSEEI MKSGEMDSVV EATEVPADIL DQPDQQDDMT QGASQAADAA

ATALAAQS

**Nup100pΔC**

MFGNNRPMFG GSNLSFGSNT SSFGGQQSQQ PNSLFGNSNN NNNSTSNNAQ SGFGGFTSAA

GSNSNSLFGN NNTQNNGAFG QSMGATQNSP FGSLNSSNAS NGNTFGGSSS MGSFGGNTNN

AFNNNSNSTN SPFGFNKPNT GGTLFGSQNN NSAGTSSLFG GQSTSTTGTF GNTGSSFGTG

LNGNGSNIFG AGNNSQSNTT GSLFGNQQSS AFGTNNQQGS LFGQQSQNTN NAFGNQNQLG

GSSFGSKPVG SGSLFGQSNN TLGNTTNNRN GLFGQMNSSN QGSSNSGLFG QNSMNSSTQG

VFGQNNNQMQ INGNNNNSLF GKANTFSNSA SGGLFGQNNQ QQGSGLFGQN SQTSGSSGLF

GQNNQKQPNT FTQSNTGIGL FGQNNNQQQQ STGLFGAKPA GTTGSLFGGN SSTQPNSLFG

TTNVPTSNTQ SQQGNSLFGA TKLTNMPFGG NPTANQSGSG NSLFGTKPAS TTGSLFGNNT

ASTTVPSTNG LFGNNANNST STTNTGLFGA KPDSQSKPAL GGGLFGNSNS NSSTIGQNKP

VFGGTTQNTG LFGATGTNSS AVGSTGKLFG QNNNTLNVGT QNVPPVNNTT QNALLGTTAV

PSLQQAPVTN EQLFSKISIP NSITNPVKAT TSKVNADMKR

**Nup2p**

MAKRVADAQI QRETYDSNES DDDVTPSTKV ASSAVMNRRK IAMPKRRMAF KPFGSAKSDE

TKQASSFSFL NRADGTGEAQ VDNSPTTESN SRLKALNLQF KAKVDDLVLG KPLADLRPLF

TRYELYIKNI LEAPVKSIEN PTQTKGNDAK PAKVEDVQKS SDSSSEDEVK VEGPKFTIDA

KPPISDSVFS FGPKKENRKK DESDSENDIE IKGPEFKFSG TVSSDVFKLN PSTDKNEKKT

ETNAKPFSFS SATSTTEQTK SKNPLSLTEA TKTNVDNNSK AEASFTFGTK HAADSQNNKP

SFVFGQAAAK PSLEKSSFTF GSTTIEKKND ENSTSNSKPE KSSDSNDSNP SFSFSIPSKN

TPDASKPSFS FGVPNSSKNE TSKPVFSFGA ATPSAKEASQ EDDNNNVEKP SSKPAFNLIS

NAGTEKEKES KKDSKPAFSF GISNGSESKD SDKPSLPSAV DGENDKKEAT KPAFSFGINT

NTTKTADTKA PTFTFGSSAL ADNKEDVKKP FSFGTSQPNN TPSFSFGKTT ANLPANSSTS

PAPSIPSTGF KFSLPFEQKG SQTTTNDSKE ESTTEATGNE SQDATKVDAT PEESKPINLQ

NGEEDEVALF SQKAKLMTFN AETKSYDSRG VGEMKLLKKK DDPSKVRLLC RSDGMGNVLL

NATVVDSFKY EPLAPGNDNL IKAPTVAADG KLVTYIVKFK QKEEGRSFTK AIEDAKKEMK

**Nup85p**

MTIDDSNRLL MDVDQFDFLD DGTAQLSNNK TDEEEQLYKR DPVSGAILVP MTVNDQPIEK

NGDKMPLKFK LGPLSYQNMA FITAKDKYKL YPVRIPRLDT SKEFSAYVSG LFEIYRDLGD

DRVFNVPTIG VVNSNFAKEH NATVNLAMEA ILNELEVFIG RVKDQDGRVN RFYELEESLT

VLNCLRTMYF ILDGQDVEEN RSEFIESLLN WINRSDGEPD EEYIEQVFSV KDSTAGKKVF

ETQYFWKLLN QLVLRGLLSQ AIGCIERSDL LPYLSDTCAV SFDAVSDSIE LLKQYPKDSS

STFREWKNLV LKLSQAFGSS ATDISGELRD YIEDFLLVIG GNQRKILQYS RTWYESFCGF

LLYYIPSLEL SAEYLQMSLE ANVVDITNDW EQPCVDIISG KIHSILPVME SLDSCTAAFT

AMICEAKGLI ENIFEGEKNS DDYSNEDNEM LEDLFSYRNG MASYMLNSFA FELCSLGDKE

LWPVAIGLIA LSATGTRSAK KMVIAELLPH YPFVTNDDIE WMLSICVEWR LPEIAKEIYT

TLGNQMLSAH NIIESIANFS RAGKYELVKS YSWLLFEASC MEGQKLDDPV LNAIVSKNSP

AEDDVIIPQD ILDCVVTNSM RQTLAPYAVL SQFYELRDRE DWGQALRLLL LLIEFPYLPK

HYLVLLVAKF LYPIFLLDDK KLMDEDSVAT VIEVIETKWD DADEKSSNLY ETIIEADKSL

PSSMATLLKN LRKKLNFKLC QAFM

**Caldesmon**

MDDFERRREL RRQKREEMRL EAERLSYQRN DDDEEEAARE RRRRARQERL RQKEEGDVSG

EVTEKSEVNA QNSVAEEETK RSTDDEAALL ERLARREERR QKRLQEALER QKEFDPTITD

GSLSVPSRRE VNNVEENEIT GKEEKVETRQ GRCEIEETET VTKSYQRNNW RQDGEEEGKK

EEKDSEEEKP KEVPTEENQV DVAVEKSTDK EEVVETKTLA VNAENDTNAM LEGEQSITDA

ADKEKEEAEK EREKLEAEEK ERLKAEEEKK AAEEKQKAEE EKKAAEERER AKAEEEKRAA

EERERAKAEE ERKAAEERER AKAEEERKAA EERAKAEEER KAAEERAKAE EERKAAEERA

KAEKERKAAE ERERAKAEEE KRAAEEKARL EAEKLKEKKK MEEKKAQEEK AQANLLRKQE

EDKEAKVEAK KESLPEKLQP TSKKDQVKDN KDKEKAPKEE MKSVWDRKRG VPEQKAQNGE

RELTTPKLKS TENAFGRSNL KGAANAEAGS EKLKEKQQEA AVELDELKKR REERRKILEE

EEQKKKQEEA ERKIREEEEK KRMKEEIERR RAEAAEKRQK VPEDGVSEEK KPFKCFSPKG

SSLKIEERAE FLNKSAQKSG MKPAHTTAVV SKIDSRLEQY TSAVVGNKAA KPAKPAASDL

PVPAEGVRNI KSMWEKGNVF SSPGGTGTPN KETAGLKVGV SSRINEWLTK TPEGNKSPAP

KPSDLRPGDV SGKRNLWEKQ SVEKPAASSS KVTATGKKSE TNGLRQFEKE P

**Nup1pΔN**

IQESFVPNSE RSGTPTLKKN IEPKKDKESI VLPTVGFDFI KDNETPSKKT SPKATSSAGA

VFKSSVEMGK TDKSTKTAEA PTLSFNFSQK ANKTKAVDNT VPSTTLFNFG GKSDTVTSAS

QPFKFGKTSE KSENHTESDA PPKSTAPIFS FGKQEENGDE GDDENEPKRK RRLPVSEDTN

TKPLFDFGKT GDQKETKKGE SEKDASGKPS FVFGASDKQA EGTPLFTFGK KADVTSNIDS

SAQFTFGKAA TAKETHTKPS ETPATIVKKP TFTFGQSTSE NKISEGSAKP TFSFSKSEEE

RKSSPISNEA AKPSFSFPGK PVDVQAPTDD KTLKPTFSFT EPAQKDSSVV SEPKKPSFTF

ASSKTSQPKP LFSFGKSDAA KEPPGSNTSF SFTKPPANET DKRPTPPSFT FGGSTTNNTT

TTSTKPSFSF GAPESMKSTA STAAANTEKL SNGFSFTKFN HNKEKSNSPT SFFDGSASST

PIPVLGKPTD ATGNTTSKSA FSFGTANTNG TNASANSTSF SFNAPATGNG TTTTSNTSGT

NIAGTFNVGK PDQSIASGNT NGAGSAFGFS SSGTAATGAA SNQSSFNFGN NGAGGLNPFT

SATSSTNANA GLFNKPPSTN AQNVNVPSAF NFTGNNSTPG GGSVFNMNGN TNANTVFAGS

NNQPHQSQTP SFNTNSSFTP STVPNINFSG LNGGITNTAT NALRPSDIFG ANAASGSNSN

VTNPSSIFGG AGGVPTTSFG QPQSAPNQMG MGTNNGMSMG GGVMANRKIA RMRHSKR

**Fesselin**

MIQSAAPSIP RVEVILDCSD REKEAPKSLA ERGCVDSQVE GGQSEAPPSL PSFAISSEGT

EQGEDNQHSE KDHRPLKHRA RHARLRRSES LSEKQVKEAK SKCKSIALLL TAAPNPNSKG

VLMFKKRRQR ARKYTLVSYG TGELERDEDE GEEGEVEEGD KENTFEVSLL ATSESEIDED

FFSDIDNDKK IVTFDWDSGL LEVEKKTKSG DEMQTLPETT GKGALMFARR RQRMDQITAE

QEEMKARTAH AEEQREVTVS ENFQKVSSSA YQTKEEEMLR QQPCISKSYA DVSQNDGKIV

QQNGFGVAPD TSLSFQSSEA QKAASLNRTA KPFPFGVQNR AAAPFSPTRN VTSPLSDLPA

PPPYCSISPP PEALYRPLSA PAASKAAPIL WSHTEPTERI ASRDERIAVP AKRTGILQEA

KRRSTSKPMF SFKEAPKVSP NPALLSLVHN AEGKKGSGAG FESGPEEDYL SLGAEACNFM

QSQASKQKAP PPTAPKPSLK VSPAAGTPVS PVWSPAVASN KAPSFPAPAS PQAAYPAPLK

SPQYPHSPSA NPPNTLNLSG PFKGPQATLA SPNHPAKTPT TPSAGETKPF EMPPEMRGKG

AQLFARRHSR MEKYVVDSET VQANMARASS PTPSLPASWK YSSNVRAPPP VAYNPIHSPS

YPPAATKPFP KSTAATKNTK RKPKKGLNAL DIMKHQPYQL DASLFTFQPP SNKESLGIKQ

IPKLPTSKQA TSLRLPGSAS PTNVRASSVY SVPAYSSQPS FQSNASTPVN ESYTPTGYSA

FSKPESTTSS LFTAPRPKFS AKKAGVIAQE RSSGRSLSLP GKPSFISRAT SPTSPLIFQP

APDYFSKPDT AADKPGKRLT PWEAAAKSPL GLVDEAFRPQ NMQESIAANV VSAAHRKTLP

EPPDEWKQKV SYEPPGPSAS LALLGGKQPG VTSARKSSLS VSNATTQAGS QQQYAYCSQR

SQTDPDIMSM DSRSDYGLST ADSNYNPQPR GWRRPT

**References**

Adkins JN, Lumb KJ (2002) Intrinsic structural disorder and sequence features of the cell cycle inhibitor p57Kip2. Proteins Struct Funct Bioinforma 46:1–7. https://doi.org/10.1002/prot.10018

Baker JMR (2009) Structural Characterization and Interactons of the CFTR Regulatory Region (PhD Thesis)

Bouvier M, Stafford WF (2000) Probing the Three-Dimensional Structure of Human Calreticulin. Biochemistry 39:14950–14959. https://doi.org/10.1021/bi0019545

Campbell KM, Terrell AR, Laybourn PJ, Lumb KJ (2000) Intrinsic Structural Disorder of the C-Terminal Activation Domain from the bZIP Transcription Factor Fos. Biochemistry 39:2708–2713. https://doi.org/10.1021/bi9923555

Chatterjee M, Pollard TD (2019) The Functionally Important N-Terminal Half of Fission Yeast Mid1p Anillin Is Intrinsically Disordered and Undergoes Phase Separation. Biochemistry 58:3031–3041. https://doi.org/10.1021/acs.biochem.9b00217

Choi UB, McCann JJ, Weninger KR, Bowen ME (2011) Beyond the Random Coil: Stochastic Conformational Switching in Intrinsically Disordered Proteins. Struct Lond Engl 1993 19:566–576. https://doi.org/10.1016/j.str.2011.01.011

Chong PA, Ozdamar B, Wrana JL, Forman-Kay JD (2004) Disorder in a target for the smad2 mad homology 2 domain and its implications for binding and specificity. J Biol Chem 279:40707–40714. https://doi.org/10.1074/jbc.M404375200

Danielsson J, Jarvet J, Damberg P, Gräslund A (2002) Translational diffusion measured by PFG-NMR on full length and fragments of the Alzheimer Aβ(1–40) peptide. Determination of hydrodynamic radii of random coil peptides of varying length. Magn Reson Chem 40:S89–S97. https://doi.org/10.1002/mrc.1132

Danielsson J, Liljedahl L, Bárány-Wallje E, et al (2008) The Intrinsically Disordered RNR Inhibitor Sml1 Is a Dynamic Dimer. Biochemistry 47:13428–13437. https://doi.org/10.1021/bi801040b

Denning DP, Patel SS, Uversky V, et al (2003) Disorder in the nuclear pore complex: The FG repeat regions of nucleoporins are natively unfolded. Proc Natl Acad Sci 100:2450–2455. https://doi.org/10.1073/pnas.0437902100

Denning DP, Uversky V, Patel SS, et al (2002) The Saccharomyces cerevisiae Nucleoporin Nup2p Is a Natively Unfolded Protein*. J Biol Chem 277:33447–33455. https://doi.org/10.1074/jbc.M203499200

Donaldson L, Capone JP (1992) Purification and characterization of the carboxyl-terminal transactivation domain of Vmw65 from herpes simplex virus type 1. J Biol Chem 267:1411–1414. https://doi.org/10.1016/S0021-9258(18)45957-1

Gall C, Xu H, Brickenden A, et al (2007) The intrinsically disordered TC-1 interacts with Chibby via regions with high helical propensity. Protein Sci Publ Protein Soc 16:2510–2518. https://doi.org/10.1110/ps.073062707

Geething NC, Spudich JA (2007) Identification of a Minimal Myosin Va Binding Site within an Intrinsically Unstructured Domain of Melanophilin*. J Biol Chem 282:21518–21528. https://doi.org/10.1074/jbc.M701932200

Goldgur Y, Rom S, Ghirlando R, et al (2007) Desiccation and Zinc Binding Induce Transition of Tomato Abscisic Acid Stress Ripening 1, a Water Stress- and Salt Stress-Regulated Plant-Specific Protein, from Unfolded to Folded State. Plant Physiol 143:617–628. https://doi.org/10.1104/pp.106.092965

Guez V, Nair S, Chaffotte A, Bedouelle H (2000) The Anticodon-binding Domain of Tyrosyl-tRNA Synthetase:  State of Folding and Origin of the Crystallographic Disorder. Biochemistry 39:1739–1747. https://doi.org/10.1021/bi992382v

Haaning S, Radutoiu S, Hoffmann SV, et al (2008) An Unusual Intrinsically Disordered Protein from the Model Legume Lotus japonicus Stabilizes Proteins in Vitro*. J Biol Chem 283:31142–31152. https://doi.org/10.1074/jbc.M805024200

Habchi J, Mamelli L, Darbon H, Longhi S (2010) Structural Disorder within Henipavirus Nucleoprotein and Phosphoprotein: From Predictions to Experimental Assessment. PLOS ONE 5:e11684. https://doi.org/10.1371/journal.pone.0011684

Hemmings H, Nairn A, Aswad D, Greengard P (1984) DARPP-32, a dopamine- and adenosine 3’:5’-monophosphate-regulated phosphoprotein enriched in dopamine-innervated brain regions. II. Purification and characterization of the phosphoprotein from bovine caudate nucleus. J Neurosci 4:99–110. https://doi.org/10.1523/JNEUROSCI.04-01-00099.1984

Kapłon TM, Rymarczyk G, Nocula-Ługowska M, et al (2008) Starmaker exhibits properties of an intrinsically disordered protein. Biomacromolecules 9:2118–2125. https://doi.org/10.1021/bm800135m

Karlin D, Longhi S, Receveur V, Canard B (2002) The N-Terminal Domain of the Phosphoprotein of Morbilliviruses Belongs to the Natively Unfolded Class of Proteins. Virology 296:251–262. https://doi.org/10.1006/viro.2001.1296

Khaymina SS, Kenney JM, Schroeter MM, Chalovich JM (2007) Fesselin is a Natively Unfolded Protein. J Proteome Res 6:3648–3654. https://doi.org/10.1021/pr070237v

Krishnan VV, Lau EY, Yamada J, et al (2008) Intramolecular Cohesion of Coils Mediated by Phenylalanine–Glycine Motifs in the Natively Unfolded Domain of a Nucleoporin. PLOS Comput Biol 4:e1000145. https://doi.org/10.1371/journal.pcbi.1000145

Langridge TD, Tarver MJ, Whitten ST (2014) Temperature effects on the hydrodynamic radius of the intrinsically disordered N-terminal region of the p53 protein. Proteins 82:668–678. https://doi.org/10.1002/prot.24449

Longhi S, Receveur-Bréchot V, Karlin D, et al (2003) The C-terminal Domain of the Measles Virus Nucleoprotein Is Intrinsically Disordered and Folds upon Binding to the C-terminal Moiety of the Phosphoprotein*. J Biol Chem 278:18638–18648. https://doi.org/10.1074/jbc.M300518200

Lowry DF, Stancik A, Shrestha RM, Daughdrill GW (2008) Modeling the accessible conformations of the intrinsically unstructured transactivation domain of p53. Proteins Struct Funct Bioinforma 71:587–598. https://doi.org/10.1002/prot.21721

Lynch WP, Riseman VM, Bretscher A (1987) Smooth muscle caldesmon is an extended flexible monomeric protein in solution that can readily undergo reversible intra- and intermolecular sulfhydryl cross-linking. A mechanism for caldesmon’s F-actin bundling activity. J Biol Chem 262:7429–7437

Magidovich E, Orr I, Fass D, et al (2007) Intrinsic disorder in the C-terminal domain of the Shaker voltage-activated K+ channel modulates its interaction with scaffold proteins. Proc Natl Acad Sci 104:13022–13027. https://doi.org/10.1073/pnas.0704059104

Mayor U, Günter Grossmann J, Foster NW, et al (2003) The Denatured State of Engrailed Homeodomain under Denaturing and Native Conditions. J Mol Biol 333:977–991. https://doi.org/10.1016/j.jmb.2003.08.062

McCubbin WD, Kay CM, Lane BG (1985) Hydrodynamic and optical properties of the wheat germ Em protein. Can J Biochem Cell Biol 63:803–811. https://doi.org/10.1139/o85-102

Neira JL, Román-Trufero M, Contreras LM, et al (2009) The Transcriptional Repressor RYBP Is a Natively Unfolded Protein Which Folds upon Binding to DNA. Biochemistry 48:1348–1360. https://doi.org/10.1021/bi801933c

Paleologou KE, Schmid AW, Rospigliosi CC, et al (2008) Phosphorylation at Ser-129 but not the phosphomimics S129E/D inhibits the fibrillation of alpha-synuclein. J Biol Chem 283:16895–16905. https://doi.org/10.1074/jbc.M800747200

Paz A, Zeev-Ben-Mordehai T, Lundqvist M, et al (2008) Biophysical Characterization of the Unstructured Cytoplasmic Domain of the Human Neuronal Adhesion Protein Neuroligin 3. Biophys J 95:1928–1944. https://doi.org/10.1529/biophysj.107.126995

Perez RB, Tischer A, Auton M, Whitten ST (2014) Alanine and proline content modulate global sensitivity to discrete perturbations in disordered proteins. Proteins Struct Funct Bioinforma 82:3373–3384. https://doi.org/10.1002/prot.24692

Permyakov SE, Millett IS, Doniach S, et al (2003) Natively unfolded C-terminal domain of caldesmon remains substantially unstructured after the effective binding to calmodulin. Proteins Struct Funct Bioinforma 53:855. https://doi.org/10.1002/prot.10481

Poznar M, Hołubowicz R, Wojtas M, et al (2017) Structural properties of the intrinsically disordered, multiple calcium ion-binding otolith matrix macromolecule-64 (OMM-64). Biochim Biophys Acta BBA - Proteins Proteomics 1865:1358–1371. https://doi.org/10.1016/j.bbapap.2017.08.019

Sánchez-Puig N, Veprintsev DB, Fersht AR (2005a) Binding of Natively Unfolded HIF-1α ODD Domain to p53. Mol Cell 17:11–21. https://doi.org/10.1016/j.molcel.2004.11.019

Sánchez-Puig N, Veprintsev DB, Fersht AR (2005b) Human full-length Securin is a natively unfolded protein. Protein Sci Publ Protein Soc 14:1410–1418. https://doi.org/10.1110/ps.051368005

Sivakolundu SG, Nourse A, Moshiach S, et al (2008) Intrinsically Unstructured Domains of Arf and Hdm2 Form Bi-molecular Oligomeric Structures In Vitro and In Vivo. J Mol Biol 384:240–254. https://doi.org/10.1016/j.jmb.2008.09.019

Soragni A, Zambelli B, Mukrasch MD, et al (2008) Structural Characterization of Binding of Cu(II) to Tau Protein. Biochemistry 47:10841–10851. https://doi.org/10.1021/bi8008856

Uversky VN, Gillespie JR, Millett IS, et al (1999) Natively unfolded human prothymosin alpha adopts partially folded collapsed conformation at acidic pH. Biochemistry 38:15009–15016. https://doi.org/10.1021/bi990752+

Uversky VN, Li J, Souillac P, et al (2002a) Biophysical Properties of the Synucleins and Their Propensities to Fibrillate: INHIBITION OF α-SYNUCLEIN ASSEMBLY BY β- AND γ-SYNUCLEINS*. J Biol Chem 277:11970–11978. https://doi.org/10.1074/jbc.M109541200

Uversky VN, Permyakov SE, Zagranichny VE, et al (2002b) Effect of Zinc and Temperature on the Conformation of the γ Subunit of Retinal Phosphodiesterase:  A Natively Unfolded Protein. J Proteome Res 1:149–159. https://doi.org/10.1021/pr0155127

Więch A, Rowińska-Żyrek M, Wątły J, et al (2019) The intrinsically disordered C-terminal F domain of the ecdysteroid receptor from Aedes aegypti exhibits metal ion-binding ability. J Steroid Biochem Mol Biol 186:42–55. https://doi.org/10.1016/j.jsbmb.2018.09.008

Wollenhaupt J, Henning LM, Sticht J, et al (2018) Intrinsically Disordered Protein Ntr2 Modulates the Spliceosomal RNA Helicase Brr2. Biophys J 114:788–799. https://doi.org/10.1016/j.bpj.2017.12.033

Yarawsky AE, English LR, Whitten ST, Herr AB (2017) The Proline/Glycine-Rich Region of the Biofilm Adhesion Protein Aap Forms an Extended Stalk that Resists Compaction. J Mol Biol 429:261–279. https://doi.org/10.1016/j.jmb.2016.11.017

Yi S, Boys BL, Brickenden A, et al (2007) Effects of Zinc Binding on the Structure and Dynamics of the Intrinsically Disordered Protein Prothymosin α:  Evidence for Metalation as an Entropic Switch. Biochemistry 46:13120–13130. https://doi.org/10.1021/bi7014822

Yiu C-PB, Beavil RL, Chan HYE (2006) Biophysical characterisation reveals structural disorder in the nucleolar protein, Dribble. Biochem Biophys Res Commun 343:311–318. https://doi.org/10.1016/j.bbrc.2006.02.153

Zeev-Ben-Mordehai T, Rydberg EH, Solomon A, et al (2003) The intracellular domain of the Drosophila cholinesterase-like neural adhesion protein, gliotactin, is natively unfolded. Proteins Struct Funct Bioinforma 53:758–767. https://doi.org/10.1002/prot.10471

Zhang X, Perugini MA, Yao S, et al (2008) Solution Conformation, Backbone Dynamics and Lipid Interactions of the Intrinsically Unstructured Malaria Surface Protein MSP2. J Mol Biol 379:105–121. https://doi.org/10.1016/j.jmb.2008.03.039
